# Unraveling Degradation Processes and Strategies for Enhancing Reliability in Organic Light-Emitting Diodes

**DOI:** 10.3390/nano13233020

**Published:** 2023-11-25

**Authors:** Syed Muhammad Kazim Abbas Naqvi, Mirza Fahad Baig, Tanveer Farid, Zahid Nazir, Syed Agha Hassnain Mohsan, Zhe Liu, Wanqing Cai, Shuai Chang

**Affiliations:** 1 School of Materials Science & Engineering, Beijing Institute of Technology, Beijing 100081, China; kazimnaqvi@bit.edu.cn; 2 Platform for Applied Nanophotonics, Faculty of Materials Science, Shenzhen MSU-BIT University, Shenzhen 518115, Chinawqcai@smbu.edu.cn (W.C.); 3 Department of Physics, Muhammad Nawaz Sharif University of Engineering & Technology, Multan 66000, Pakistan; fahadmirza0031@gmail.com; 4 School of Chemical Engineering, Nanjing University of Science and Technology, Nanjing 210094, China; tanveer_af@yahoo.com; 5 Optical Communication Laboratory, Ocean College, Zhejiang University, Zheda Road 1, Zhoushan 316021, China; hassnainagha@zju.edu.cn; 6 Beijing Engineering Research Center of Mixed Reality and Advanced Display School of Optics and Photonics, Beijing Institute of Technology, Beijing 100081, China; 3220215084@bit.edu.cn

**Keywords:** analytical techniques, degradation, failure modes, organic light-emitting diodes (OLEDs), lifetime

## Abstract

Organic light-emitting diodes (OLEDs) have emerged as a promising technology for various applications owing to their advantages, including low-cost fabrication, flexibility, and compatibility. However, a limited lifetime hinders the practical application of OLEDs in electronic devices. OLEDs are prone to degradation effects during operation, resulting in a decrease in device lifetime and performance. This review article aims to provide an exciting overview of OLED degradation effects, highlighting the various degradation mechanisms. Subsequently, an in-depth exploration of OLEDs degradation mechanisms and failure modes is presented. Internal and external processes of degradation, as well as the reactions and impacts of some compounds on OLED performance, are then elucidated. To overcome degradation challenges, the review emphasizes the importance of utilizing state-of-the-art analytical techniques and the role of these techniques in enhancing the performance and reliability of OLEDs. Furthermore, the review addresses the critical challenges of lifetime and device stability, which are crucial for the commercialization of OLEDs. This study also explores strategies to improve OLEDs’ lifetime and stability, such as using barrier layers and encapsulation techniques. Overall, this article aims to contribute to the advancement of OLED technology and its successful integration into diverse electronic applications.

## 1. Introduction

**Figure 1:**
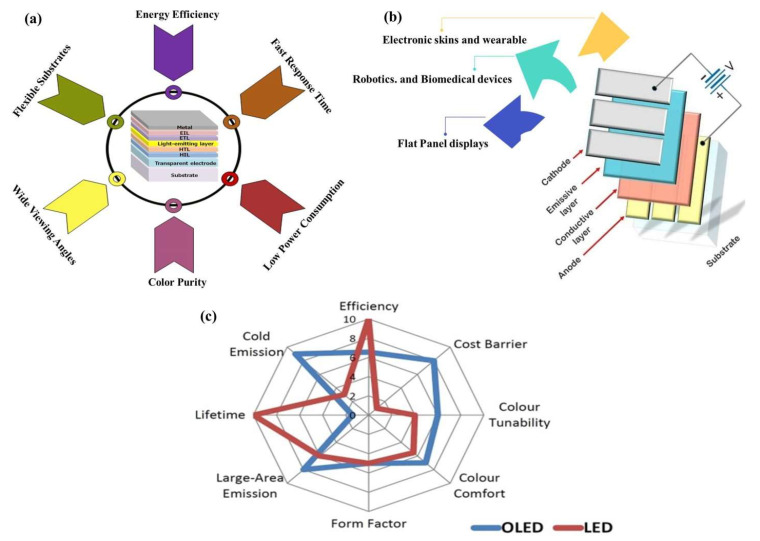
(**a**) Advantages of OLED. (**b**) Applications of OLED. (**c**) Comparison of OLED and LED. Reprinted from “The Global Information Hub for Lighting Technologies and Design”.

Organic light-emitting diodes (OLEDs) are poised to become the leading technology in high-quality flat-panel display [1] and solid-state lighting. Their disruptive features, including energy efficiency [2], wide viewing angles, fast response times, high contrast, and exceptional color purity (Figure 1a), make them ideal candidates for various applications (a few of them are shown in Figure 1b). In contrast, traditional LEDs require complex processing to achieve comparable light quality, and achieving uniform dispersion into a near-plane light source remains a challenge for them. A clear comparison of LEDs and OLEDs is shown in Figure 1c.

**Figure 2:**
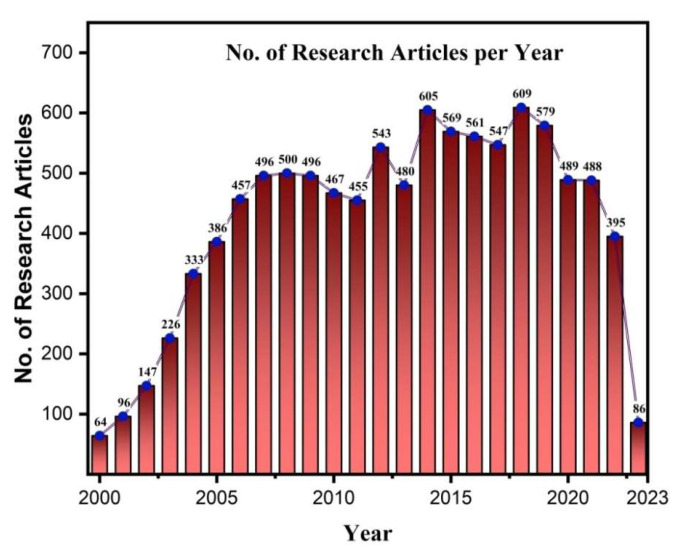
No. of research articles published per year till now on OLED (Web of Science).

**Figure 3:**
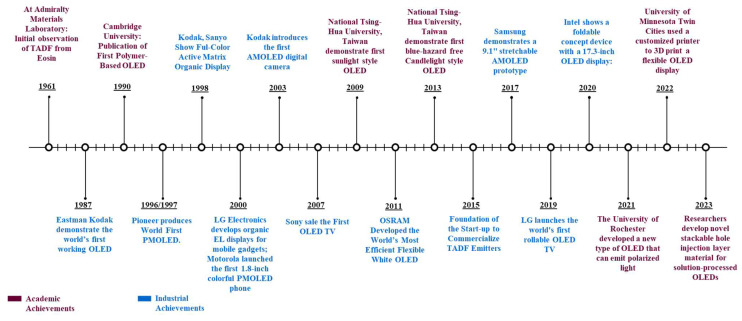
Academic and industrial achievements of OLED.

Moreover, OLED technology offers unique advantages in flexible and stretchable substrates [3], enabling applications in wearable electronics [4], biomedical devices [5], electronic skins [6], and robotics [7]. OLEDs’ thinness, flexibility, and durability allow them to withstand harsh mechanical conditions, such as bending and twisting. Additionally, OLEDs offer the ability to adjust color and color temperature over a wide range, allowing for the replication of natural light styles. While OLEDs excel in various applications, their use in general lighting presents cost challenges. The manufacturing costs of components increase proportionately when larger OLED lighting panels are required for uniform illumination in spaces like offices or reading areas. Due to their importance in industry and academia, OLEDs are receiving much focus in research with a large number of research articles published every year on the topic, shown in Figure 2 and Figure 3.

While OLED researchers and market analysts are optimistic about OLED technology disrupting conventional lighting and capturing a significant market share, it is crucial to streamline market-related content. The Global OLED Display Market, valued at 43,066 million USD in 2022, is projected to reach 11,709,730 million USD by 2028, with a CAGR of 18.14%. This growth is driven by the rising adoption of OLED technology, advancements in display technology, and increasing consumer demand for high-quality displays.

Researchers are exploring innovative strategies to enhance efficiency and mitigate degradation in organic light-emitting diodes (OLEDs) [8]. A notable approach involves the utilization of thermally activated delayed fluorescence (TADF) materials, capable of converting less efficient triplet excitons into more efficient singlet excitons. This holds the potential for significant improvements in OLED efficiency [9]. Another avenue is the adoption of triplet–triplet annihilation (TTA), a process wherein two triplet excitons combine to form a singlet exciton, contributing further to enhanced OLED efficiency. Despite some limitations of TADF OLEDs, such as broad emission spectra and longer lifetimes, the technique of hyperfluorescence proves effective in overcoming these constraints [10]. Hyperfluorescence entails employing TADF emitters as assistant hosts for fluorescent end-emitters, resulting in OLEDs with up to 100% internal quantum efficiency (IQE) [11]. The introduction of TED-TADF, a novel OLED technology amalgamating the advantages of TADF and TTA, demonstrates the efficient harnessing of triplet excitons and their conversion into singlet excitons, leading to heightened device efficiency and prolonged operational life, particularly in deep-blue OLEDs [12,13]. Overall, numerous promising approaches to enhancing OLED efficiency, including TADF, TTA, and TED-TADF, are showing significant potential, with ongoing research aimed at refining techniques for further improvements.

Despite the numerous advantages, one significant challenge that OLEDs face is degradation during operation, which can lead to a decrease in device lifetime and performance. OLED degradation mechanisms can occur internally or be influenced by external factors. Internal degradation processes involve reactions within the OLED materials, such as the degradation of organic layers or the formation of non-radiative recombination sites. One of the most common intrinsic degradation mechanisms in OLEDs is the formation of excitons (electron–hole pairs) in the organic layers. These excitons can generate reactive species, such as free radicals and triplet excitons, which react with organic materials, forming non-emissive species and reducing device efficiency [14,15]. Additionally, the diffusion of organic molecules from the emissive layer into adjacent layers or the substrate can result in the formation of non-emissive species or chemical reactions with other materials in the device. Metal ions diffusing from the cathode into the organic layers can also contribute to reduced efficiency [16]. External factors, such as moisture, oxygen, and UV radiation exposure, can also contribute to OLED degradation. Moisture and oxygen can react with organic materials and affect their properties, impacting efficiency and lifetime. Light exposure can generate reactive species like singlet oxygen, which can also lead to the formation of non-emissive species [17]. OLED degradation can also occur through various factors, including chemical, electrical, thermal, and optical degradation. Chemical degradation is caused by exposure to environmental factors like oxygen and moisture, leading to reduced efficiency [18]. Electrical degradation occurs due to charge buildup in the organic layers, resulting in chemical reactions and device degradation [19]. Thermal degradation occurs when OLEDs are exposed to high temperatures, degrading organic materials and decreasing efficiency [20,21,22]. Optical degradation occurs when OLEDs are exposed to light, causing degradation of the organic materials [23].

Understanding and mitigating these degradation issues are crucial for OLED technology’s commercialization and widespread adoption. Researchers and industry experts are actively exploring strategies to improve the lifetime and stability of OLEDs. Strategies have been proposed to mitigate degradation, and researchers are actively developing methods to improve the stability and durability of organic materials, optimize device structures, and enhance fabrication processes. One approach is to enhance the stability of organic materials through modifications in chemical structure or the addition of stabilizing additives [24]. Using phosphorescent iridium complexes as dopants can reduce the formation of reactive species [25]. Small molecule stabilizers like tetraphenylethene (TPE) can also suppress non-emissive species formation [26]. Improved encapsulation using barrier layers like metal oxides or polymers can protect OLEDs from external factors [27,28]. Studies have proposed techniques such as using interlayers to block exciton migration [29] and optimizing device structures for better thermal stability [30]. While progress has been made, further research is needed to develop more robust and durable OLED devices. Efforts are also underway to enhance the understanding of degradation mechanisms by utilizing state-of-the-art analytical techniques. These techniques enable researchers to study the degradation processes in real-time and identify the underlying causes. By gaining a deeper understanding of degradation mechanisms, researchers can develop strategies to mitigate these issues and improve OLEDs’ overall performance and reliability. Despite the challenges posed by degradation, the market potential for OLED technology remains promising. This review paper summarizes the recent research on the degradation mechanisms in OLEDs and the strategies to mitigate their impact. In summary, OLED degradation involves a combination of intrinsic and extrinsic factors. Understanding these mechanisms and implementing strategies to minimize degradation is essential for enhancing the performance and lifetime of OLED devices.

## 2. The Lifetime of OLEDs

OLEDs may undergo internal decay through various mechanisms like chemical reactions, morphological changes, and physical changes like charge accumulation. These mechanisms can impact the device’s color, luminance, current, and voltage properties, which may eventually reduce the device’s efficiency and lifetime. The luminance behavior of OLEDs over time typically assessed at a constant physical environment such as temperature, current density, voltage, or humidity, is the most critical factor in determining the device’s lifetime. The device lifetime is usually expressed as T_50_ or T_1/2_, the duration for the luminance to dim to half its initial brightness at a constant current density [31]. More complex metrics such as T_70_ and T_97_ measure how long it takes for the luminance to drop to 70% and 97% of its initial value, respectively. The T_97_ metric is significant for the display industry as it measures the threshold at which humans can perceive differences in brightness between adjacent display elements. Although some researchers have assessed device lifetime using constant voltage sources, this can cause luminance drops to occur more quickly as the device’s impedance rises with time, decreasing current density.

Interestingly, the luminance of OLEDs often varies directly with the current density across a wide range. This suggests that as the current density delivered to the OLED increases, its lifetime typically decreases, according to the well-known empirical scaling law $L_{0}^{n}⸱T_{1/2}=C$ [31,32,33]. In fact, researchers have found they can predict the lifetime of very stable OLEDs with reasonable accuracy by studying the relationship between a device’s initial luminance at time zero (L_0_) and its lifetime (T_1/2_), using a constant factor (C), as described in studies by Meerheim et al. and Jarikov et al. [34,35]. The rate of degradation of a device over time can be influenced by the acceleration factor (n), which is specific to the materials and design of the device under study. However, accurately predicting the lifetime of very stable organic devices can be challenging due to the potential for inaccuracies [33,34]. In some cases, it can be difficult to predict an OLED’s actual lifetime since various factors can cause degradation. Researchers may observe an initial sharp decline in luminance followed by a more gradual, long-term decline. To account for these complex patterns, researchers use a combination of different exponential decay functions, such as LtL0=ae−αt+be−βt. This equation includes several fitting parameters, including a, b, α, and β, which can be used to predict the OLED’s lifetime more precisely [33]. The alternative method for predicting the lifetime of OLEDs is through the stretched exponential decay (SED) function, which is represented by, $\frac{L_{t}}{L_{0}}=exp[-{\left(\frac{t}{\tau }\right)}^{\beta }]$ [33,34,36]. The degradation of OLEDs is linked to the formation of defects within the device, which can function as luminescent quenchers, nonradiative recombination centers, or deep charge traps based on their energy levels [37,38]. It has been found that at 50% luminance loss in a particular system, the areal density of fixed charges linked with these defects is around 6 × 10^11^ cm^−2^ (or 10^−7^ C/cm^2^) in size [39]. Giebink et al. investigated electroluminescence loss utilizing a mathematical model with various arbitrary factors, which showed that a defect density of 10^18^ cm^−3^ (about 0.1% of the molecular density) might cause a loss of more than 50% of the device luminance [38]. However, it is crucial to remember that these equations might underestimate or overestimate the lifetime if inadequate data were utilized for fitting [34,40]. The SED function has two fitting parameters, τ and β, representing the decay constant and stretching factor, respectively, but neither holds any specific physical significance. It has been empirically found that β varies for different material configurations and stack designs but remains the same for various current densities of identical OLED stacks.

**Figure 4:**
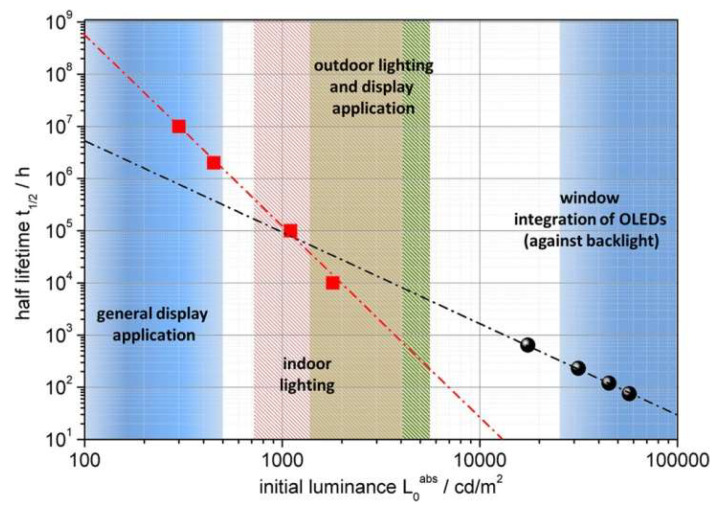
Overview of relevant OLED brightness, needed for several applications. Current lifetime data are from the state-of-the-art red (red ■) and black (●) OLEDs from Meerheim et al. and Löser et al., respectively [34,40].

The way lifetime data are reported and compared has changed over the last few years, in line with the criteria for device application by industrial standards. An overview of relevant OLED brightness, needed for several applications is shown in Figure 4. Initially, device lifetime was commonly reported at an expected display brightness of 100 cd/m^2^, but, nowadays, the majority of lifetime data are published as luminance, which is 1 order of magnitude higher, i.e., 1000 cd/m^2^, suitable for both simple lighting applications and demanding display applications. It is well-known that indoor displays usually have an overall brightness ranging from 100 to 300 cd/m^2^ [41]. To attain this range of brightness, individual pixels on the device must emit light at levels between 200 and 600 cd/m^2^, depending on the pixel’s size, color, and device architecture [32,42]. In 2001, Howard and Orache from IBM and Chwang et al. from UDC predicted that a display would need to last for over 10,000 h at 140 cd/m^2^, assuming continuous operation and integration into devices such as entertainment headsets [36,43]. The incorporation of filters such as polarizers in displays also increases the device lifetime requirements. According to the same IBM study, even modest outdoor displays may require brightness as high as 3000 cd/m^2^. This requirement extends to the industry and governmental bodies, as evidenced by the German Federal Ministry of Education and Research project to achieve a lifetime of 30,000 h for white OLEDs at 1000 cd/m^2^.

**Figure 5:**
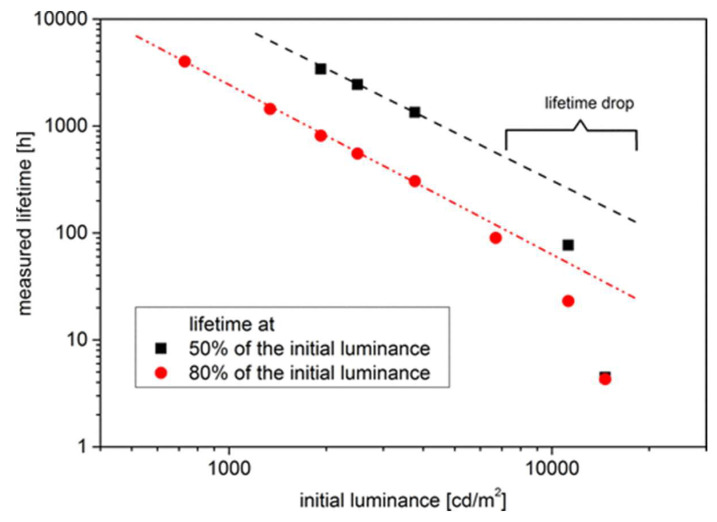
The measurement of lifetime at varying initial luminances highlights the inadequacy of lifetime extrapolation due to the amplified Joule heating of a red OLED. This figure depicts the lifetime data at 80% (T80) and 50% (T50) of the initial luminance, with lines provided as a visual aid. Reprinted from ref. [44]. Copyright 2014 Orgworld.

Developing stable organic devices is a challenge, as they are prone to temperature-enhanced degradation. Researchers have been exploring acceleration tests to understand this problem better and develop more accurate lifetime models. Figure 5 shows the lifetime measurement at varying initial luminance, depicting the lifetime data at 80% (T_80_) and 50% (T_50_) of the initial luminance. One such test is the 80/80/80 test, which involves determining the device lifetime under conditions of 80 °C, 80% humidity, and after reaching 80% of its initial luminance. These tests consider the device’s increased degradation behavior under these conditions and can provide valuable insights into the underlying physics of degradation. By understanding the acceleration of degradation, researchers can work towards improving the overall stability and lifetime of organic devices.

## 3. OLEDs Failure Modes

### 3.1. Catastrophic Failure

When a device fails, it is usually due to electrical short circuits, the most severe type of failure. These shorts occur due to imperfections on the surface of the device’s substrate, such as particles, rough surfaces, or local heating during operation [45,46,47,48]. The heat generated by these imperfections can also cause structural changes in the device, potentially leading to electrode contact or damage to the cathode material [49,50,51]. In severe cases, these changes may result in the complete malfunction of the entire OLED. Occasionally, the electrodes may separate, leading to gas accumulation from the decomposition of materials, resulting in dark spots or bubbles [51].

**Figure 6:**
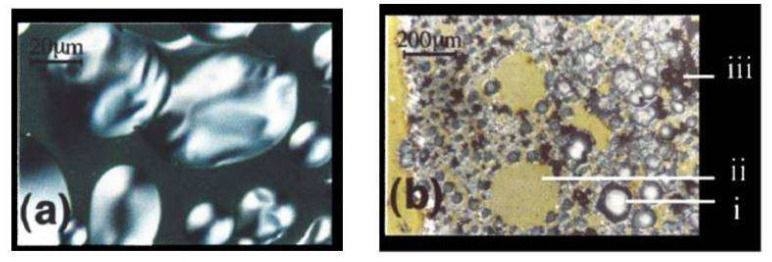
Microphotographs of the device under external illumination: (**a**) Viewed through the metal electrode side and showing the ‘‘bubbles’’ at the delamination stage; big bubbles are typically 40–80 mm in diameter. (**b**) Viewed through the ITO glass substrate after the failure and displaying various damage patterns: (**i**) bubble about 100 mm diameter; (**ii**) peeling area with black spots concentrated on its perimeter, and (**iii**) black spots—carbonized areas [52].

Research on the specifics of “catastrophic failure” has been limited, as the processing of devices has dramatically improved, typically preventing this type of degradation. However, investigations into polymeric OLEDs revealed changes in voltage and luminance measurements changes just before device failure [52]. These fluctuations are thought to be caused by the growth of dark spots and bubbles as illustrated in Figure 6, eventually leading to cathode delamination and complete breakdown of the device [52]. Another possible explanation for catastrophic failure is the electromigration of the cathode metal during device operation, resulting in thin metal filaments spreading through organic layers or even reaching the opposite electrode [52,53]. This strong dependence of this mechanism on current density and temperature, the role of pinhole defects in initiation filament growth, and the self-healing behavior presumably brought on by filaments’ periodic formation and destruction make it difficult to distinguish it from other failure modes. In the latest OLED devices, electromigration still plays a significant role in catastrophic failures and leakage currents, such as those observed below the turn-on voltage.

### 3.2. Dark Spot

In the active region of OLEDs, dark spots, also known as black spots, are regions that do not emit light and emerge under specific operating or storage conditions. It has been discovered that dark spots can form at the boundaries between the organic and conducting layers and expand in response to electrical stress [54,55,56,57,58,59]. Research by McElvain and Aziz has demonstrated that an increase in dark spots can result in reduced OLED efficiency, brightness, and lifetime [60,61]. Various factors contribute to the appearance of dark spots, such as humidity, dust particles, pinholes, spikes on ITO, and short circuits. An analysis of the mechanisms by Azrain and colleagues in 2018 identified pinholes and high electrical current density as the main causes of dark spots in OLEDs [62]. According to Weijer and colleagues, local oxidation of the cathode due to water penetration through pinholes can create non-emissive portions of the device [63]. To predict the development of black spots in flexible OLEDs, Ohzu and colleagues have developed a method based on quantifying the amount of water entering the device [64]. Ding and others have addressed the features of catastrophic OLED lighting panel failure and its relevance to the growth of dark spots [65].

In the 1990s, researchers proposed encapsulation as a preventive measure against dark spots forming, aiming to extend the device’s lifetime. According to Ke et al., dark spots can appear due to metal migration roughening the polymer/electrode interface, leading to increases in local current. Smoothing the polymer/electrode interface was recommended to minimize this effect [56]. Chan et al. and Phatak et al. observed that the formation of black dots could be avoided by a temperature increase during cathode layer deposition by increasing the adhesion between the organic and cathode layer [66,67]. According to research by Liu et al., base etching can smooth the anode (ITO) surface without affecting its thickness or sheet resistance, extending the OLED’s lifetime and avoiding the occurrence of dark spots [68]. To prevent cathode delamination and dark spot formation, Liew et al. also suggested a novel technique of removing the cathode using scotch tape and installing a substitute cathode in a vacuum chamber [69].

**Figure 7:**
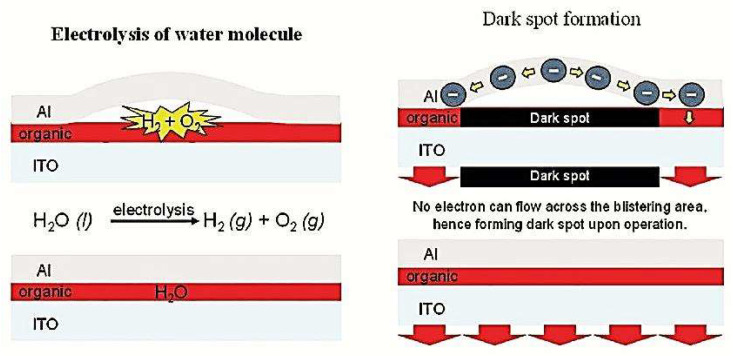
Electrolysis of water molecules and formation of dark spots inside an OLED device [44].

By reducing the number of dark spots, the performance of the OLED device can be enhanced, particularly in terms of brightness, efficiency, and lifetime. A diagram illustrating the mechanism of dark spot formation due to electrolysis of water molecules is shown in Figure 7. Overall, studies on the growth of dark spots in OLEDs have improved our understanding of the factors contributing to device degradation, prompting efforts to enhance OLED design and manufacturing procedures to prevent the development of dark spots.

### 3.3. Substrate Fracture

Excessive heat generation inside OLED devices poses a significant challenge that can lead to device meltdown or substrate fracture. According to Chen et al., one of the primary issues faced by flexible OLEDs is the breakage of very thin and brittle conducting transparent oxide films, which depend on the deposition temperature [70]. The production of Joule heat ultimately limits the lifetime of OLEDs, leading to interlayer diffusion of materials, thermal expansion, and the crystallization or melting of organic materials [71]. However, there is a potential solution to this problem as research conducted by Yan et al. developed an organic electronics substrate made of polycarbonate that can withstand high temperatures without melting or deforming [72].

Current research efforts focus on developing innovative materials, structures, and encapsulation methods to protect OLEDs from the challenges caused by high heat. Technological advancements have enabled OLED development with improved efficiency, reduced power consumption, and longer lifetimes. For instance, by employing phosphorescent materials instead of fluorescent ones, the efficiency of OLEDs has increased up to four times. Additionally, solution-processed OLEDs, which can be produced using inexpensive printing techniques, show promise for the mass manufacturing of flexible OLED displays.

### 3.4. Luminance Decay

The lifetime of an OLED is generally measured by the period it takes for the luminance to decrease to 50% of its base value [73]. Several factors, including coulombic decay [74], accumulation of OLED degradation products, high temperatures [75], and the proliferation of immobile charge carriers, influence this decrease in luminance [76,77,78]. Wang et al. categorized the decay stages into three categories to better understand the decay behavior [79]. Additionally, Aziz et al. identified dark-spot degradation, catastrophic failure, and intrinsic deterioration as the main causes of OLED luminance loss [48]. Ishii et al. explained that luminance decay occurs in two phases, exponential decay due to chemical degradation and a quick downward movement due to an internal electric field [80]. Young et al. discovered that NPB+ can suppress blue luminescence by Forster energy transfer [81].

Current research focuses on finding solutions to address the problems caused by luminance degradation in OLEDs [82]. One solution is using novel materials that can withstand high temperatures and avoid the accumulation of OLED degradation products, immobile charge carriers, and traps [79]. Another strategy is to improve the overall design and construction of OLED devices to prevent or minimize the impact of these factors. For example, Lee et al. have improved luminance efficiency by using OLEDs with tandem device structures and adding hole-transporting interlayers [83]. Kitamura et al. added SiO_2_/SiN_x_ photonic crystals to an ITO substrate to increase luminance efficiency, while Li et al. added CNT templates as an external electron source to compensate for the device’s lack of electrons and increase luminance efficiency [84,85]. Microlens arrays have also been discovered as an effective way to increase brightness efficiency in OLED displays [86].

**Figure 8:**
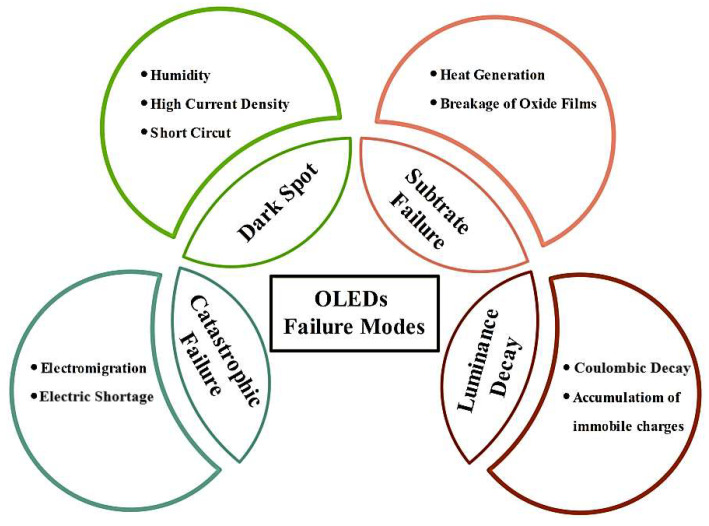
Failure modes of OLEDs.

In summary, the lifetime of an OLED is determined by the period it takes for the luminance to decrease to 50% of its base value, which is influenced by several factors shown in Figure 8. Current research focuses on finding solutions to address the problems caused by luminance degradation in OLEDs [87], including using novel materials and improving the design and construction of OLED devices. Various strategies have been proposed to enhance luminance efficiency, including the use of tandem device structures [88], hole-transporting interlayers [89], SiO_2_/SiN_x_ photonic crystals, CNT templates, and microlens arrays [90].

## 4. Degradation Mechanism in OLEDs

OLEDs, like all electronic devices, are subject to degradation over time, which can harm their performance and longevity. Understanding the factors contributing to the degradation in OLEDs is crucial for improving their stability and expanding their range of applications. Various factors, such as exposure to environmental conditions, aging of organic materials, and inefficiencies in the light-emitting process, can contribute to OLED degradation. Performance issues resulting from OLED degradation include black spots, altered colors, decreased efficiency, and catastrophic failures. Exposure to oxygen and moisture can cause the oxidation and degradation of organic materials [91], leading to a drop in efficiency, and brightness, and an increase in operating voltage, eventually resulting in device failure. Organic materials used in OLED fabrication are susceptible to thermal, photo, and chemical degradation over time, decreasing efficiency and brightness. Poor charge transport or light extraction can also result in reduced brightness and efficiency, increasing the operating voltage and eventually leading to device failure. Material selection, device structure, and operating conditions can influence the degradation rate. In the pursuit of optimizing OLED performance, researchers have explored the impact of external pressure on device reliability. Lee et al. [92] showcased improvements in luminance and current efficiency under pressure. Tang et al. [93] reported a significant boost in quantum yield and device current efficiency, emphasizing the potential for external pressure to enhance molecular aggregation fluorescence in 2008. However, a comprehensive examination of overall device stability was lacking until recently.

**Figure 9:**
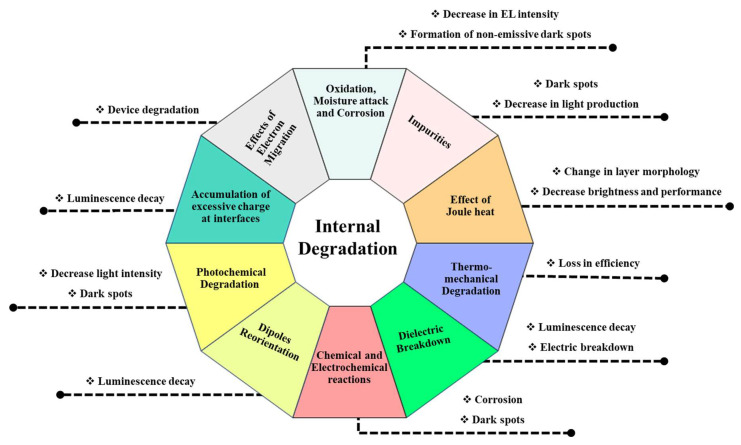
Internal degradation processes.

**Figure 10:**
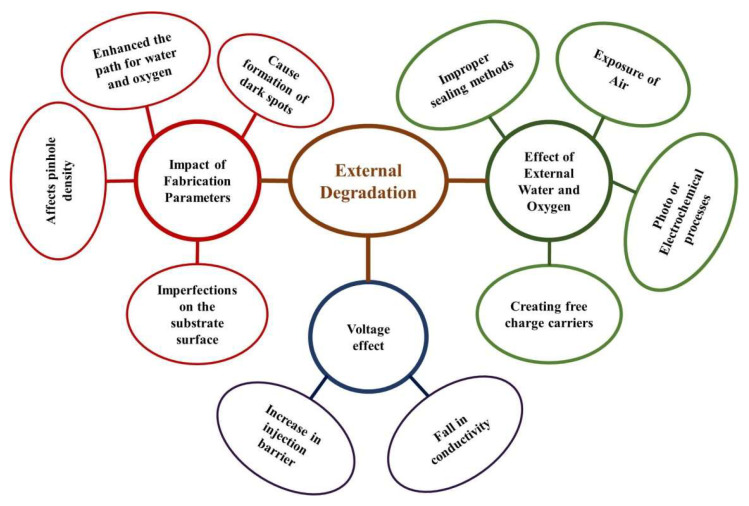
External degradation processes.

Extensive research is necessary for a better understanding of OLED degradation processes (summarized in Figure 9 and Figure 10) and failure mechanisms, and further improvements in the performance and stability of OLEDs. Ongoing research and development are critical to address these challenges and enhance the lifetime and competitiveness of OLEDs. In conclusion, understanding OLED degradation processes and failure mechanisms is crucial for improving their stability and lifetime.

### 4.1. Internal Causes of Degradation

#### 4.1.1. Oxidation, Moisture Attack, and Corrosion

OLED technology faces a significant challenge due to the impact of oxidation on device performance and lifetime. Moisture is one of the main contributors to OLED device degradation, which has a variety of adverse effects and significantly reduces the device’s lifetime. For example, it may cause dark spots to appear and spread, which can be undesirable and affect the OLED’s function. Additionally, moisture can cause a decrease in electroluminescence intensity, hindering the OLED’s ability to emit light. Moreover, it may modify the organic layers’ electrical structure, further degrading the device’s functioning. The range of adverse effects can vary from reduced performance to complete device failure.

Water vapors can damage the organic materials and electronic components inside the device’s encapsulation layers by penetrating the layers and giving rise to a serious threat to OLEDs. One way moisture can impact OLEDs is via hydrolysis, which causes the device’s organic components to break down and lose their capacity to emit light. The OLED’s performance is continually affected by the chemical processes that occur, which change according to the materials used in the device. Moisture can also corrode the metallic contacts and other components in the OLED, leading to issues like electrical shorts, open circuits, and other forms of damage. This can be especially concerning because corrosion can be challenging to detect, and significant damage may have already occurred when the issue is identified.

Furthermore, moisture provides an ideal environment for the growth of microorganisms, which can lead to additional damage to the OLED and potential health hazards for users. It is evident that safeguarding OLEDs from moisture should be a top priority for anyone working with these devices. Corrosion of the cathode is another potential consequence of moisture exposure, which can cause electrical shorts and other problems. It is particularly challenging to protect OLEDs from moisture because they can penetrate through materials.

Corrosion of the cathode is another potential consequence of moisture exposure, which can cause electrical shorts and other problems. One approach to figuring out how much moisture may penetrate a material is the damp heat (DH) test, which is described in the “61215 Test” by the International Electrotechnical Commission (IEC) [94]. Researchers have discovered that grid corrosion or reduced conductivity between emitter and grid resulted from DH-induced degradation. This kind of corrosion can have severe consequences for OLEDs, as it can cause significant damage to the device [95]. Similar studies have looked at the degradation of photovoltaic modules by corrosion, highlighting the necessity to address this issue in various devices [96,97]. Burrows and colleagues have pointed out that oxygen and water can dramatically damage the OLED device lifetime [98]. Researchers have investigated how moisture affects the intensity of OLEDs’ electroluminescence [96]. Their results demonstrate that moisture damages the HTL and ETL layers, which can lead to the formation of dark spots, operational instability, and ultimately device degradation. Exposure to moisture causes changes in the tris-(8-hydroxyquinoline) aluminum (Alq3) layer’s electrical structure [99]. In a related study, researchers found that Alq3 degradation occurs in the presence of moisture and oxygen [100]. Other studies have also verified an electrolytic reaction caused by moisture that has become adsorbent on the electrode surface [101].

The fiber white OLED is encapsulated by an Al_2_O_3_/elastomer bilayer, showing stable operation under repetitive bending and pressure, and in water. This pioneering work is believed to provide building blocks for realizing complete textile display technologies by complementing the lack of the core technology [102]. A silane-based inorganic–organic hybrid layer (silamer) was introduced onto Al_2_O_3_ films to enhance their environmental stability. This resulted in the preservation of Al_2_O_3_ film characteristics through the formation of a robust and continuous aluminate phase of Al-O-Si at the interface, particularly in hygrothermal environments [82].

According to Lim et al., pinholes can increase the susceptibility to moisture attacks by providing an undesirable penetration channel, leading to the development of dark spots. These studies and findings highlight the importance of protecting OLEDs from moisture and corrosion to maintain their performance and extend their lifetime. Developing improved encapsulation techniques and enhancing device design ensures that OLEDs are better equipped to withstand moisture impact and maintain optimal functionality. Oxidation in OLEDs is caused by electrochemical reactions and moisture attacks, leading to the formation of non-emissive dark spots and a reduced device lifetime [60]. Researchers have been working to address these problems since the development of the first organic electroluminescent device [103]. Studies have shown that oxidation can reduce OLED materials’ carrier mobility and induce defects during fabrication [104]. The thermal diffusion of oxygen also promotes the oxidation of organic layers and electrodes. To slow down oxidation, moisture control is a viable solution [105]. Device encapsulation can effectively stop oxidation, and researchers have explored the use of metallic capping layers and encapsulation as potential solutions. Various practical approaches have been identified to reduce the risk of moisture attack, including encapsulation [106]. The feasibility of a moisture seal, which utilizes alternate layers of organic and inorganic materials, such as epoxy and SiNH, has been discussed, as well as the use of different types of desiccants to prevent moisture attacks. By effectively preventing moisture penetration, the performance and lifetime of the OLED device can be significantly increased.

Corrosion is another major problem affecting the performance and lifetime of OLED devices [107]. Corrosion processes in OLEDs can affect the electrodes or organic/polymer layers, ultimately impacting the device’s lifetime. Researchers have found that galvanic corrosion can lead to bubble formation in OLEDs, and bases can enhance the corrosion of polymers and the formation of bubbles under electrical stress conditions [108]. In flexible optoelectronics, ITO is easily subjected to stress corrosion cracking, mainly when acids are present [109]. The corrosion rate can be reduced using efficient encapsulating technologies. Utilizing substrates with low penetration rates for air and water is key to minimizing the corrosion rate of different layers in OLEDs [110]. The extent of moisture penetration is frequently determined using the calcium corrosion test, and scientists have invented a gold-doped Mg cathode to stop corrosion-related non-emissive area development [111,112].

#### 4.1.2. Effects of Electron Migration on OLED

There has been extensive research on metals migrating when subject to DC electric fields and currents in the microelectronics and electroceramics industries [113,114]. Silver (Ag) is especially prone to this behavior, especially when humidity is present [113,115]. The oxidation of Ag at the anode into Ag^+^ cations, which subsequently migrate through the electric field and are electrochemically reduced back to zerovalent metal when they come into contact with the cathode, is one proposed mechanism [115]. Some systems are mainly produced by momentum transfer from moving charge carriers to metal atoms, which are subsequently pushed forward by an “electron wind” or “hole wind” [116,117]. To distinguish it from electrochemical migration, this latter mechanism is called electromigration. Organic light-emitting diodes (OLEDs) and photovoltaic cells are two examples of organic optoelectronic devices that typically include silver in their electrodes. To lower the resistive losses of transparent electrodes with insufficient intrinsic conductivities, it is employed as a continuous sheet, an Ag nanowire network, or shunting lines. Hence, short-circuiting caused by electrically driven Ag migration may be a factor in the short lifetimes of organic optoelectronic devices with Ag in one or both electrodes.

The collision of electrons with each other results in molecular migration and a loss of device efficiency [118,119]. Shen et al. claim that mobile ions induce voltage fluctuations that lead to device degradation [120]. The presence of indium in the organic layer and interactions between the metal and organic layers can enhance metal migration [121] and decrease luminance efficacy [122]. Small organic molecules like TPBi can commence migration even at a low voltage bias [123], and Png et al. noticed PEDOT^+^ accumulation at the contact between PEDOT:PSS and HIL [124]. Recent studies have explored various strategies to address these challenges and enhance OLED performance [125]. Li et al. developed a new technique to build a highly stable OLED with reduced exciton quenching and higher efficiency by employing metal-free organic emitters [126]. Pan et al.’s study addressed this gap by revealing that the proper application of pressure enhanced charge carrier mobility and increased photoluminescence intensity. A 10 min application of 0.75 MPa pressure improved current efficiency by over 12% and significantly increased device stability, extending the half-decay lifetime from 245 to 431 min [127]. The challenges faced by flexible OLEDs in terms of operational stability and mechanical degradation are acknowledged. To address these issues, achieving a balanced charge-transport and injections, along with the use of flexible encapsulation methods such as Flex Lami-capsulation, becomes essential [128]. Additionally, Zhang et al. enhanced the efficiency and stability of blue OLEDs by utilizing a thermally activated delayed fluorescence material as an emitting layer, achieving a peak external quantum efficiency of 32.8% [129]. Several studies have explored the utilization of alternative materials and device designs such as tandem OLEDs for enhanced efficiency and color purity, along with organic–inorganic hybrid perovskite halide materials [130].

#### 4.1.3. Photochemical Degradation

OLED technology relies on organic and polymeric materials, which are prone to reactions affecting device performance [131,132,133]. One indication of a photochemical reaction is the release of oxygen from the indium tin oxide (ITO) electrode when excitons migrate toward it after being absorbed by the polymer layer [131]. Scholz et al. have investigated this process in three critical steps. On the other hand, excitons play a crucial role in the deterioration of OLED devices through electrochemical reactions [134]. More specifically, they drive intramolecular cyclization in the hole-transporting layer (HTL) [135]. Moreover, their interaction with positive polarons can accelerate device degradation at the interface between the hole transport material (HTM) and the electron transport material (ETM) [136]. UV radiation-induced singlet excitons can also accelerate device degradation. This effect can be mitigated by adding a hole-blocking layer to control the exciton distribution [137]. To further understand the mechanism of photochemical reactions in OLED devices, researchers have employed laser-desorption/ionization time-of-flight mass spectrometry (LDI-TOF-MS) [95]. UV exposure can cause photochemical degradation in OLED devices, resulting in dim or dark spots [138], chemical bond breakage in the CBP component [139], reduced light emission capability [140], decreased light intensity [141], and singlet exciton generation that damages chemical bonds [142]. To prevent this damage, minimizing exposure to UV light is essential. Frequent UV exposure contributes to device degradation and the age of reactive oxygen species [54]. Additionally, exposure to UV light can decrease the efficiency of white OLED devices by damaging the light-emitting layer [143]. Strategies to mitigate the effects of UV light include the use of novel UV filter materials [144].

The research investigates the degradation mechanism of doped blue fluorescent OLEDs based on TTA hosts, revealing that increased aging time results in elevated triplet monomolecular decay rate (kT) and reduced bimolecular TTA up-conversion rate (γTTA). This indicates that device degradation is primarily attributed to damaged TTA molecules and weakened TTA up-conversion. The insertion of a TCTA thin layer between the EML and the ETL successfully enhances OLED lifetime from 46 to 151 h by reducing the density of excess charges, offering a theoretical basis for optimizing the longevity of TTA-based blue fluorescent OLEDs [145]. Exciton-induced processes also significantly degrade OLED devices. Kim and co-workers have investigated the formation of polaron pairs from dopant to host excitons [146]. The diffusion of excitons from the HTL to the emissive layer (EML) and the formation of quenchers in the HTL also contribute to device degradation [118]. Aziz et al. have conducted numerous experiments to assess the impact of excitons on OLED efficiency and lifetime. This includes the study of exciton-induced degradation on HTLs and its impact on the EQE and lifetime of phosphorescent OLEDs [147]. They found that introducing HTL can limit electron leakage to the hole injection layer (HIL) and enhance device stability. Aziz et al. also investigated the morphological characteristics of spin-coated and blade-coated organic semiconductor films [148]. The study showed that devices with HTL and EML blade coatings were less aggregated and had greater efficiency and lifetime. They also studied exciton-induced molecular aggregation in organic small-molecule electroluminescent materials and found that solution-processed thin films were more susceptible to aggregation than vacuum-deposited ones. Lower deposition rates improved morphological stability and order while reducing the impact on PhOLED EL performance and thin-film morphology [149].

#### 4.1.4. Accumulation of Excessive Charge at Interfaces

OLED devices may experience an accumulation of excessive charge at interfaces, which results in nonradiative recombination centers and luminescence quenchers. Research indicates that fixed charges are responsible for the accumulation within devices, particularly in proximity to the interface, which also serves as a charge recombination zone [150]. Charges and excitons have a significant impact on OLED degradation, but the accumulating effects can be minimized by combining materials for hole and electron transport in the emission zone, resulting in lower excitons and local densities of charge [151]. Various approaches have been proposed to address the charge accumulation issue. These include enhancing the quality of interfaces between different layers and incorporating innovative materials with improved stability, such as electron transport materials. Researchers are developing new encapsulation techniques to shield OLEDs from elements that can cause damage [152]. One most promising technique is using flexible and transparent barrier materials that can protect OLEDs from oxygen and moisture while retaining excellent optical transparency [153]. The optimization of carrier accumulation at the hole-transporter/emission layer interface results in impressive characteristics, including a narrow emission spectrum, high color purity, and an extended operational lifetime [154]. The device lifetime of green phosphorescent OLEDs was improved by mitigating electron accumulation at the EBL/EML interface, achieved through optimizing the device structure, leading to an increased operating lifetime from 233 h to 399 h at 1000 cd/m^2^ [155]. Researches have investigated hybrid OLEDs with solution-processed ZnO layers, achieving an exceptional measured lifetime (LT_95_) of 875 h by passivation of the ZnO surface with a thin Al layer. This highlights the potential of solution-processed ZnO-based inverted OLEDs for cost-effective large-scale manufacturing in future display and lighting applications [156]. Through advanced device engineering, particularly in reducing carrier accumulation at the hole-transporter/emission layer interface, the optimized device achieved impressive characteristics: a narrow full width at half maximum of 32 nm, CIE coordinates of (0.28, 0.67), external quantum efficiency of approximately 19%, and a remarkable lifetime (LT_50_) of 14,000 h at 1000 cd/m^2^ [157]. The incorporation of Yb within the charge generation layer (CGL) significantly enhances the performance and lifetime of series OLEDs. The tandem OLED demonstrates an extended lifetime (T_90_) of 308 h, outperforming single-unit OLED S (T_90_ = 49 h) by 6.3 times [158].

#### 4.1.5. Dipole Reorientation

Certain theories propose that molecules with permanent dipole moments undergo reorientation when exposed to electric fields of 1 MV/cm in amorphous substances [159,160]. The reorientation of molecules in OLED layers can have implications for the electric polarization of the layer, potentially influencing the overall geometry of the device’s electric field. This, in turn, can impact various processes such as outcoupling efficiency, quenching of excited states by charge carriers, and recombination. Luminance decay, which represents a distinct degradation mechanism, can be influenced by all of these factors. As reorientation is reversible, it is anticipated that this degradation mechanism is also reversible. Molecular dipole reorientation could be a substantial element in OLED degradation and was first sparked by the early observation of partial recovery of brightness efficiency [159]. Yet, molecule dipole reorientation could likely inadvertently contribute to OLED deterioration, particularly during the initial stages of the degradation process.

#### 4.1.6. Chemical and Electrochemical Reactions

The degradation of OLEDs can be significantly impacted by chemical and electrochemical reactions, resulting in irreversible damage [161]. To ensure long-lasting OLEDs, Xia et al. suggest that developing electrochemically stable EML is critical since it comprises electron and hole charge carriers [162]. Aziz et al. reported that short circuits can accelerate the degradation process, while electrochemical reactions between the electrodes can lead to corrosion and microstructural abnormalities [163]. Potential factors in device degradation include the instability of Alq_3_ cations and anodic oxidation of tris(8-hydroxyquinoline)aluminum (Alq_3_) [164]. The formation of H_2_ gas-filled “bubbles” due to moisture at the metal/organic interface can lead to the formation of dark spots and local luminescence [52,56,77,165].

To improve device lifetime, Rudmann et al. proposed using more electrochemically stable metals, such as Ag, as the cathode [166]. Recent studies have further investigated the effects of electrochemical reactions on OLED degradation. To extend the lifetime of carbene–metal–amide (CMA) OLEDs, minimizing exciton–exciton annihilation is crucial. Strategies include flattening the recombination zone, improving electron transport in the emissive layer, increasing layer thickness, and designing deep-blue light emitters, with a need for more stable CMA variants focused on stabilizing the excited state or reducing susceptibility to degradative exciton–exciton interactions [167]. Wu et al. found that hydroxide ions are produced during the electrochemical reaction between indium tin oxide (ITO) and water, which can severely impact OLEDs [168]. According to their study, the electrochemical stability of OLEDs can be improved by a thicker ITO layer. A study explores subatomic modification of TADF emitters via targeted deuteration, revealing increased photostability with higher deuterated substitutions. Deuteration of both donor and acceptor extends OLED device lifetime from 15 h to 23 h at 1000 cd/m^2^ luminance, showcasing the potential of subatomic-level modifications in enhancing operational stability across OLED applications [169]. Gao et al. demonstrated that the electrochemical stability of OLEDs can be significantly increased by reducing the formation of radical species by using a lithium salt additive [170]. Yuan et al. also reported the potential of novel materials and techniques, such as the application of electrochemically stable materials, the addition of shielding layers, and interface modification, to improve the electrochemical stability of OLEDs [171]. Three new bipolar host molecules (1-,2-,and 4-3cbzBIZ) with superior thermal properties were introduced into OLEDs, resulting in a high-performance blue OLED (max η_EQE_ of 28.6%) and a green OLED with both higher efficiency and extended operational lifetime (11 times improvement at 80 °C) attributed to the exceptional thermal stability of 4-3cbzBIZ [172].

#### 4.1.7. Dielectric Breakdown

While there are limited studies on the dielectric properties of OLED materials, dielectric breakdown is often associated with electric breakdown in OLEDs. Researchers have suggested that using an electron injection layer with high dielectric strength can improve the efficiency and brightness of devices [173]. A study showcases the fabrication of unitary metal–dielectric photonic crystal (MDPC) OLEDs with varying layers, using alternating Ag:Al and Ag:Mg alloy mirrors to control the photonic band structure with insights into the roles of extinction coefficient and refractive index [174]. Furthermore, the influence of material dielectric strength on active matrix OLEDs has been explored, revealing that the substrate’s roughness can impact the breakdown of the dielectric properties of organic materials [175]. Phosphotungstic acid (PWA), leveraging its negative differential resistance (NDR) property, prevents electrical leakages in OLEDs on uneven aluminum-foil substrates, offering a low-cost solution for robust luminescence in flexible states, applicable to diverse thin-film devices for advancing the Internet of Things [176]. Although the standard driving voltage for OLED devices is typically between 3 and 5 V [177], higher voltage is necessary for brighter displays. However, the luminance may begin to decline once the voltage surpasses 10 V, primarily due to the dielectric breakdown of organic materials [44].

#### 4.1.8. Thermo-Mechanical Degradation

OLED devices experience stress because the layers of various materials have a unique thermal expansion coefficient [111,178]. According to Lee et al., this tension is discharged by a procedure known as delamination [111]. Delamination can also be caused by mechanical loads such as bending, twisting, and folding [111]. The thermo-mechanical properties of the materials utilized significantly influence the lifetime and efficiency of flexible OLEDs [179]. Brand et al. emphasized the importance of matching the secondary materials in the device’s elastic moduli, adhesion strength, and coefficient of thermal expansion [180]. Poly(benzoxazoleimides) (PBOI), a super heat-resistant polymer substrate with a very low coefficient of thermal expansion and excellent ductility for flexible OLED devices, was introduced by Hasegawa et al. in 2017 [181]. According to Oh et al., flexible OLEDs performed much better when fabricated of polyethylene naphthalate (PEN), which has a low coefficient of thermal expansion (13 ppm °C). Newly synthesized oxetane-containing materials demonstrated superior hole transporting properties in OLEDs, exhibiting low turn-on voltages, high luminous efficiencies, and significant brightness, showcasing their potential in optoelectronics, particularly when combined with an additional PEDOT layer for enhanced device performance [182]. Efficient blue OLEDs with high-temperature resistance were achieved by introducing charge injection layers (SILs) TAPC:50 wt% 9,10-di(naphth-2-yl)anthracene (ADN) and TPBi:50 wt% ADN, improving carrier balance and maintaining a steady recombination position for excitons under thermal stress [183]. Novel Pt(II) complexes with tetradentate coordination and donor–acceptor structure demonstrate exceptional performance in red OLEDs, featuring high efficiency (EQEs of 31.8%, 26.8%, 30.8%), good color purity, low driving voltage, and an operational lifetime exceeding 14,000 h at 1000 cd/m^2^ [184].

#### 4.1.9. Effect of Joule Heat

OLED devices’ thermal instability is a significant concern for their long-term performance as it can cause degradation. Electronic devices produce heat when in use due to the resistance in their circuits. This heat, known as Joule heat, can have several detrimental effects and reduce the device’s lifetime, brightness inhomogeneity, and performance [22,185,186,187]. Researchers have utilized techniques like scanning tunneling microscopy and photoluminescence to study the impact of Joule heat on the interfacial structure and phase separation of various materials. High localized current in devices has been observed to increase Joule heat [188], which can cause the release of gases, resulting in the formation of bubbles within OLED devices. Besides Joule heating, significant heating in practical devices like screens and luminaires is also attributed to voltage drops on ITO:metal electric bus lines and TFT circuits. At high temperatures, several vital reactions, like changes in layer morphology and phase transitions of materials, may occur in addition to the internal accelerated response. It should be noted that temperature rises can come from a variety of causes, such as outside influences or Joule heating, and they may have an overall effect or only be limited to device flaws. At lower driving currents, the effect of morphology on device performance can be neglected. It is only at higher current densities that produce significant Joule heating or when the device is subjected to high temperatures that the effect of morphology becomes noticeable. Doped transport layers can be employed to reduce Joule heating, which lower voltage and increase the overall device resistance [189]. An innovative methodology utilizing crystalline organic single-crystal (OSS) thin films was introduced to fabricate high-performance C-OLEDs, achieving a maximum EQE of 6.5% and surpassing reported high-EQE deep-blue amorphous OLEDs in driving voltage, power efficiency, and structural stability under challenging environments [190].

#### 4.1.10. Impurities

The purity of materials used in Organic Light-Emitting Diodes (OLEDs) significantly impacts their brightness and lifetime. The amount of impurities in organic materials affects the performance of Organic Semiconductor Devices (OSCs) and OLEDs. Researchers have shown that the use of highly pure materials can improve the flow of electricity through OLEDs, leading to increased light production and enhanced device performance and longevity [159,191]. However, certain impurities, such as halogenated compounds, can damage specific components of OLEDs. Dust particles on OLED surfaces can also create tiny holes that allow air and moisture to enter, potentially damaging the device [62]. Furthermore, impurities inside the vacuum chamber can affect device longevity and reproducibility [192]. Therefore, researchers use techniques such as HPLC-MS and CIC to detect such impurities [193].

According to Liao et al. [194] during the annealing process of glass or ITO substrate at 200 °C, impurities and moisture can quickly diffuse, acting as nonradiative recombination centers or catalysts for the dissociation processes of metal-organic emitters. Highly pure materials and ultra-high vacuum conditions can significantly extend the lifetime of OLEDs. Xia et al. demonstrated the importance of employing highly refined materials by increasing the lifetime of a device by changing the dopant purity from 99.8% to 100%, according to HPLC analysis [195]. Researchers have discovered that using a new chamber can result in a device lifetime that is twice as long as using an old one [196]. Tsugita et al. used a gas flow deposition process to synthesize high-quality organic films and demonstrated an effective mass spectrometric technique for identifying internal impurities in OLED displays [197,198]. Surface treatments such as mechanical polishing, annealing, and etching have been introduced to modify the surface roughness of Indium Tin Oxide (ITO) [199]. Choi et al. found that boron doping can alter the surface characteristics of ITO, and Zhou et al. discovered that sandblasting techniques could modify the wave nature of internally generated photons, improving the external quantum efficiency [200]. Silane-modified ITO surfaces have been shown to have higher power efficiency than regular ITO surfaces by adjusting the anode work function to the HOMO of HTL, according to Hatton et al. [201]. Kim et al. found that the OLED leakage current is directly correlated with the peak-to-valley roughness of the ITO surface [202]. Plasma treatment has been reported to minimize surface roughness and contamination, according to Park et al. and Lu et al. [203]. Lastly, chlorinating transparent ITO enhances the electrode work function, as observed by Helander et al. [204].

### 4.2. External Processes of Degradation

It is important to consider external and internal factors that impact the device to define the causes and mechanisms of OLED degradation properly. External factors like oxygen and water can considerably affect OLEDs’ lifetime [91]. The performance of OLEDs can also be influenced by regularly occurring variations in temperature and light, some of which are known and controllable. There are still unknown aspects to take into account, though. For instance, further research is needed to fully understand how temperature affects the degradation rate and light’s impacts on OLEDs. Below the glass transition temperature, the device may undergo morphological changes that result in total failure. To increase OLEDs’ overall performance and extend their lifetime, it is crucial to understand these elements better. A summarized overview of the external degradation process is shown in Figure 10.

#### 4.2.1. Impact of Fabrication Parameters

**Figure 11:**
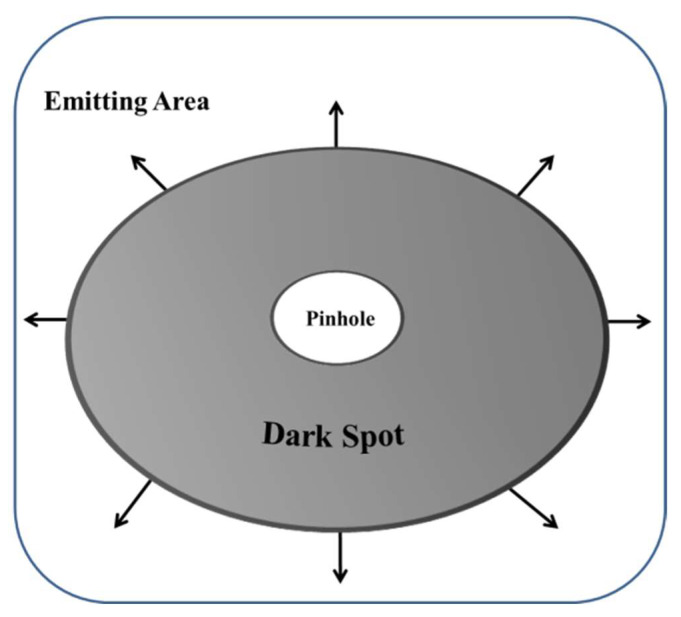
Visualization of dark spot due to pinhole.

Fabrication parameters play a significant role in the lifetime of OLED devices [205]. Imperfections on the substrate surface can influence the pinhole density in the device structure (shown in Figure 11), and the deposition rate may affect the morphology of organic layers and overall device characteristics [187,206]. In addition, studies have shown that heating the substrate during deposition can increase the device lifetime, possibly due to in situ water and oxygen desorption during the annealing process. However, the exact mechanisms behind this improvement are still not fully understood [207].

Ikeda et al. found that, while the process pressure had no significant effect on the early device performance, increasing the process pressure led to a decrease in device lifetime. The amount of water and oxygen in the process chamber can significantly influence the amount of oxygen incorporated into the device, particularly at the organic/metal interface. This can produce oxidized components that are mostly found near the organic/metal interface [208,209]. Researchers have suggested that the presence of oxygen at the organic/metal interface may be due to “hot” metal atoms interacting with the underlying organic material, especially for organic layers that contain oxygen [210]. Understanding the underlying chemical processes during the manufacture of devices is crucial to maximizing their performance and stability. In terms of degrading reactions in organic devices, water content is believed to have a greater impact than the remaining amount of oxygen. Water can generate fluorescence quenchers and electron traps, leading to an increase in driving voltage [208,211,212]. Deposition of metal oxide transparent electrodes is also a crucial process parameter to consider, especially for top-emitting OLEDs. Sputtering, a commonly used deposition technique can cause serious harm to the device due to strong particles and UV radiation. While appropriate protective layers may limit particle damage, shielding devices from strong UV radiation remains challenging [213,214]. Studies have shown that UV radiation may cause photochemical reactions, such as dimerization and fragmentation within the OLED structure, leading to a shorter device lifetime [215].

#### 4.2.2. Effect of External Water and Oxygen

Device encapsulation must be discussed first to comprehend the complexity of water and oxygen impact. Despite years of research on flexible OLED displays, traditional sealing methods still exhibit substantial moisture absorption of the plastic substrate [216]. OLEDs require transmission values orders of magnitude lower than those achievable with conventional sealing methods such as food packaging and liquid crystal displays. It has been established that the penetration rates of oxygen and water vapor correlate with the defect densities of the barrier materials, as they commonly enter electrode materials through defects at the edges of a cathode [217]. The equivalent growth rates for oxygen-induced dark patches are orders of magnitude lower than those for water-induced processes. A thermal activation energy of 0.3 eV for the development of black spots in the presence of oxygen was determined assuming Arrhenius’ law [218].

Oxygen has imperative effects on the OLED’s performance besides the formation of dark spots. A “doping” effect with oxygen sometimes causes an increase in conductivity, which is occasionally reversible by purging the device with nitrogen [60,219,220]. The device’s increased conductivity may be due to the development of a (weak) charge transfer state between the oxygen and the organic molecule [101,209] and the quenching process [220,221,222]. It is expected that an OLED’s performance will change when subjected to external factors, including oxygen and water [101,219,223]. Exposure to air has a significant impact on light output, as demonstrated by Kaminorz et al. with a polymeric OLED, despite there not being much alteration in the I-V curve (a greater current is detected) [220]. The light output was reduced by an order of magnitude when comparing devices kept under nitrogen to those kept in ambient conditions. Water is also known to cause fluorescence quenching [224]. The two components, oxygen and water, are known to react differently with various materials.

**Figure 12:**
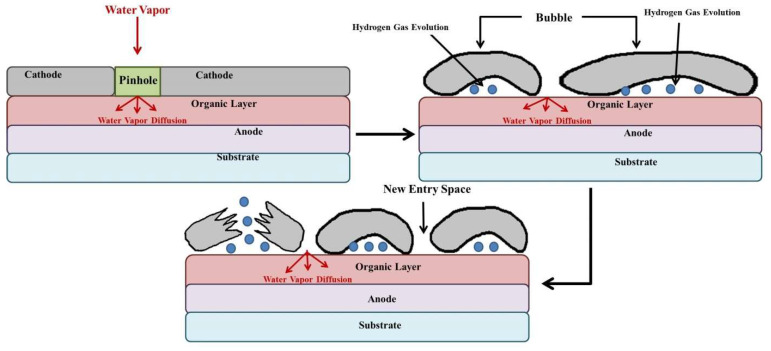
Mechanism of dark spot formation.

OLEDs are affected by oxygen and water as illustrated in Figure 12. Several substances, such as electropositive metal cathode oxidation releasing elemental hydrogen, disintegrating chelate molecules causing a variety of reactions in the end products, photo- or electrochemical processes oxidizing organic molecules, creating filled traps and/or free charge carriers from charge-transfer doping, and altering the structure of materials, including crystallization, can initiate a variety of processes that cause the damage [120,224,225,226,227,228,229].

#### 4.2.3. Voltage Effect

When OLEDs degrade, there is usually a decrease in their brightness and a notable voltage increase due to electrical resistance [120,230]. Studies suggest that this voltage increase is caused by a rise in the injection barrier at particular interfaces and a fall in the conductivity of certain transport layers. The accumulation of traps may be the origin of these changes, and various interfaces and transport layers are thought to be involved in these phenomena. Research has revealed that the voltage increase in polymer OLEDs is significantly influenced by the ITO/organic interface [231,232]. In recent studies, the decreasing ITO work function has been examined using X-ray photoelectron spectroscopy (XPS). These findings emphasize the importance of studying how ITO interacts with organic materials to enhance the functionality and stability of organic electrical devices [233].

According to Sato and Kanai, one potential cause of the voltage increase that occurs as a device is aging is the oxidation of the cathode/organic interface [207]. One strategy that has been used to address this problem is the application of calcium as a “sacrificial anode” on top of a Mg:Al cathode, resulting in the creation of a “getter” that efficiently scavenges oxygen and water, adding another layer of protection [234]. It is important to note that when the calcium layer was not in close contact with the underlying cathode, its ability to prevent the creation of dark spots was found to be less effective. Although the advantages of using a bilayer cathode structure on the device lifetime have not yet been demonstrated, it is possible that less dark-spot growth will result in a decreased voltage rise and a potential increase in the operational lifetime of the device.

## 5. Visualizing Degradation Process in OLEDs by State-of-the-Art Analytical Techniques

**Figure 13:**
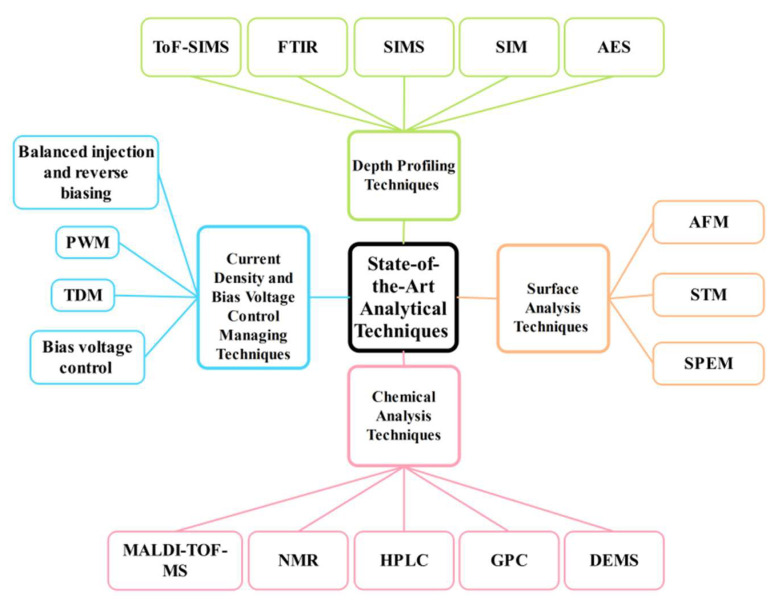
Techniques to visualize degradation in OLEDs.

Physical and chemical analytical methods for determining how organic semiconductor devices degrade are essential for assessing simple and intricate stacks of organic semiconductors used in real-world devices. A variety of physical and chemical properties, including diffusion, morphological or chemical changes, and accumulation of charge, must be considered when analyzing the causes of device deterioration. These mechanisms have been studied in recent years using various analytical techniques (summarized in Figure 13). These instruments and methods can be categorized according to how they react electrically, optically, chemically, or mechanically, and how sensitive they are to various device parameters, such as the interfaces, bulk, or surface. A suitable analytical technique is essential to comprehend a particular topic fully. Researchers have evaluated organic devices using a variety of cutting-edge analytical approaches. Through the use of these methods, we may examine and acquire essential knowledge about the ways by which organic devices degrade. In conclusion, choosing the right analytical techniques is essential for thoroughly analyzing the routes for organic devices that degrade and fail.

### 5.1. Current Density and Bias Voltage Control Managing Techniques

Organic light-emitting diodes’ current–voltage (I-V) characteristic is a fundamental characteristic that provides key information about the transport, injection, and recombination processes. It is possible to compare devices with systematically various layer thicknesses to evaluate variations in the I-V characteristic brought on by device aging.

Several electrical technologies and procedures have been developed to address OLED degradation issues. One approach is the management of current density, as excessive current densities can lead to hotspots and charge accumulation, resulting in degradation. Active matrix driving technologies, such as pulse-width modulation (PWM) and time-division multiplexing (TDM), can control current density and reduce the chances of degradation. Another strategy involves bias voltage control to minimize charge trapping and prolong the OLED’s lifetime. Techniques like balanced injection and reverse biasing can alter the bias voltage and mitigate charge buildup [235]. Various analytical methods, such as the Stark effect, charge modulation spectroscopy, thermally accelerated current spectroscopy, impedance spectroscopy, and voltametric measurements, can provide insights into trapping, transport, and injection behavior within the OLED layers. Optical techniques, such as infrared (IR) and Raman spectroscopy, ellipsometry, and microscopy techniques like photoluminescence microscopy and scanning near-field optical/atomic-force microscopy (SNOM-AFM), are employed to study molecular structure changes, doping levels, layer thicknesses, and the growth of dark-spot regions. Combining multiple spectroscopic methods offers a more comprehensive understanding of organic semiconductors’ chemical and structural transformations.

### 5.2. Surface Analysis Techniques

Surface examination of devices can provide crucial information about several aspects of degradation. Atomic force microscopy (AFM) is a common method for assessing a material’s roughness and changes, crystallization, and morphology-related lifetime properties. It can also be utilized to evaluate the outcomes of surface enhancements and the characteristics of organic material deposition. Scanning tunneling microscopy (STM), which is less frequently used, however, may evaluate molecule orientation and shape in connection to the degradation behavior of organic semiconductors. A spatially resolved technique called scanning photoelectron microscopy (SPEM) is frequently utilized to investigate localized chemical processes such as cathode delamination or dark patches. Recent studies have shown that these techniques are useful for analyzing the surface of organic semiconductors. Researchers fabricated cathode layer by co-deposition of silver (Ag) and aluminum (Al) metals using thermal evaporation [236]. They fabricated the thermally stable and high transmittance Ag:Al TC. Moreover, research has shown that employing XPS and UPS techniques to examine the surface of organic semiconductors may offer crucial information regarding the chemical degradation pathways occurring on the surface of devices. In situ photoemission spectroscopy can be employed to look at the reasons causing tandem OLEDs to degrade. These techniques highlight the value of surface analysis techniques and procedures for examining OLED deterioration causes and creating efficient mitigation plans. More dependable and long-lasting OLED devices can be fabricated for various applications by developing a more thorough grasp of the degradation mechanisms. The performance and stability of OLEDs depend greatly on the interface between organic layers. That is why this work emphasizes the value of using surface analysis techniques that can probe that contact.

### 5.3. Depth-Profiling Techniques

Depth-profiling techniques shed light on the degradation mechanisms and direct the fabrication of more resilient OLEDs. A surface-sensitive method, Time-of-flight secondary ion mass spectrometry (ToF-SIMS), was employed in a study to examine the deterioration of a blue phosphorescent OLED [237]. The findings demonstrated that the degradation began at the interfaces between the various organic layers and that, over time, the degradation products diffused into the surrounding layers. The degradation of solution-processed OLED was examined using Fourier transform infrared spectroscopy (FTIR) [238]. The results showed that the depth of the OLED influenced the degree of degradation and that it was caused by the dissolution of chemical bonds in the organic components. This knowledge could be applied to the design of OLEDs to create stronger chemical bonds or to enhance the design of the organic layers to reduce chemical bond breakage. Secondary ion mass spectrometry (SIMS) examined a blue phosphorescent OLED degradation [239]. The findings demonstrated that the organic materials were degraded due to metal ions diffusing into the organic layers from the cathode. With the help of this knowledge, OLEDs with more stable cathode materials might be created, or the thickness and organic layer composition could be adjusted to reduce metal ion diffusion. Insights into the mechanisms of degradation that can be gained from depth-profiling techniques can help to fabricate OLEDs that are more resistant to degradation.

In addition to these methods, which also include dynamic XPS depth profiling and scanning ion microscopy (SIM), scanning electron microscopy (SEM), and Auger electron spectroscopy (AES) have been used employed to explore various OLED features, including diffusion and migration processes. These methods have been employed recently to look into deterioration brought on by internal and external parameters.

### 5.4. Chemical Analysis Techniques

Understanding the chemical composition of OLEDs is one of the main difficulties in device investigations. Chemical analysis techniques are used to study the degradation of OLEDs (organic light-emitting diodes) over time. These techniques help researchers understand the chemical and physical changes that occur in OLEDs as they degrade, which can ultimately help to improve their performance and lifetime.

Only a few methods, such as matrix-assisted laser desorption/ionization time-of-flight mass spectrometry, were used in the past to provide chemical information (MALDI-TOF-MS). The fields of macro- and biochemistry are where MALDI-TOF-MS is most frequently utilized as a powerful trace analysis technique. Another frequent tool is high-performance liquid chromatography (HPLC) in combination with different detectors. Yet, the device’s component dissolution/extraction is a crucial phase that restricts sensitivity because there are few materials per unit of extractable surface in absolute terms. Furthermore, some chemical byproducts of device breakdown are anticipated to be very reactive and most likely incompatible with solvent extraction and chromatography. While gel permeation chromatography (GPC) can be successfully utilized to identify high-molecular-weight OLED products, other chromatographic techniques, such as gas chromatography (GC), are restricted to molecules with relatively low molecular weights. However, a variety of chemical analytical techniques, including differential electrochemical mass spectrometry (DEMS), nuclear magnetic resonance (NMR), and electron paramagnetic resonance (EPR), can be used to specific concerns of device degradation. Studies have focused on improving the sensitivity and specificity of these techniques, such as developing new methods for analyzing the chemical composition of OLEDs, improving the sensitivity of MALDI-TOF-MS, and combining different analytical methods for more comprehensive analysis. A strong tool for examining the chemical characteristics of OLEDs is spectroscopy. To learn more about the energy levels of the molecules involved in OLED emission, it is possible, for instance, to utilize UV-Vis spectroscopy to examine how OLED materials absorb light. Fluorescence spectroscopy can be used to study the emission properties of OLEDs, which can help identify degradation mechanisms and products. Researchers have used spectroscopy to study OLED degradation in a variety of contexts, including in the presence of oxygen, moisture, and other environmental factors. In conclusion, chemical analysis techniques are powerful tools for studying OLED degradation. Spectroscopy, chromatography, mass spectrometry, electrochemical analysis, and thermal analysis can all provide valuable insights into the chemical and physical changes that occur in OLEDs as they degrade. By understanding the degradation mechanisms and products, researchers can develop strategies to improve the performance and lifetime of OLEDs.

## 6. Key Challenges in OLEDs and Strategies for Reliability Enhancement

Despite the remarkable advancements and significant growth in the field of OLED technology in the past decade, with the introduction of the first OLED displays in the market, there are still numerous obstacles that must be addressed to establish OLED displays as a dominant player in the field of flat-panel displays (FPDs) and distinguish it from other FPD technologies. These challenges can be conveniently categorized into four distinct areas, each presenting unique difficulties and complexities. Overcoming these hurdles will require innovative solutions, further research, and development to fully realize the potential of OLED displays and position them as a top choice for consumers and industries.

There are still many challenges to counter to establish OLED displays as a dominant player in the field of flat-panel displays (FPDs) and set them apart from other FPD technologies, despite the impressive advancements and significant growth in the field of OLED technology over the past ten years, with the introduction of the first OLED displays in the market. These issues can be conveniently divided into four categories, each with special challenges and complications. To overcome these obstacles and establish OLED displays as a top option for consumers and industry, creative ideas, additional research, and developments are necessary.

### 6.1. Material-Related Challenges

Materials play a crucial role in determining the stability and performance of OLEDs, making material-related challenges a significant focal point for OLED technology. Limited material availability and stability pose hurdles, as certain OLED materials can be expensive and prone to degradation over time. Researchers are actively developing new materials that offer improved stability and scalability. Another challenge lies in the limited color gamut of OLEDs, as existing materials struggle to produce certain hues. Scientists are working on new materials with better color performance to expand the color range [240,241]. Poor charge injection and transport characteristics of OLED materials also affect device efficiency and brightness [242]. Exploring materials with enhanced charge transport capabilities, such as crystallinity-enhanced small molecules or molecularly aligned polymers. The exploration of spontaneous orientation polarization (SOP) in organic thin films, particularly in electron transport materials (ETMs) used in OLEDs, opens new avenues [243,244]. Researchers have developed a non-synthetic method to control SOP by diluting the ETM TPBi with medium-density polyethylene (MDPE). This not only significantly reduces SOP but also minimally impacts charge transport. The benefits are evident in blue fluorescent OLEDs, where eliminating SOP led to a lowered operating voltage, increased EQE by 30%, a 50% improvement in luminous efficacy, and a threefold extension of device lifetime. The reduction in exciton–polaron quenching (EPQ) in the emissive layer, thanks to a decrease in excess hole density induced by SOP, underscores the potential of controlling SOP as a valuable tool for improving OLED performance [245]. The introduction of a new class of Au(I)-TADF materials based on a carbene–metal–amide (CMA) molecular framework showcases exceptional thermal stability, intense TADF light emission spanning a wide range of colors, and impressive device lifetimes. The vacuum-deposited Au(I) OLEDs using these materials achieve exceptional brightness, high EQEs, minimal performance degradation, and remarkable device lifetimes [238]. The synthesis of deep-blue emitters with asymmetric structures (B-N-S-1/2/3) and their integration into OLEDs, especially B-N-S-3 with an extended operational lifetime, represents a promising avenue for stable blue OLEDs [239]. Molecular engineering strategies at the subatomic level offer promise in enhancing the stability of blue OLEDs. The replacement of hydrogen with deuterium (deuteration) on the acceptor unit of thermally activated delayed fluorescence emitters has significantly improved photostability and extended device lifetime. Isotopic analogues have gradually improved the operational stability of OLEDs, demonstrating the potential for further advancements in stable and durable blue OLED devices [165]. Furthermore, the toxicity of some OLED materials raises concerns for the environment and human health. Focusing on developing safer and more environmentally sustainable materials will be beneficial to address this. By overcoming these material challenges, OLED technology can continue to evolve and improve its performance.

### 6.2. Patterning Techniques

OLED patterning techniques face challenges that impact resolution, uniformity, material compatibility, cost, scalability, and alignment. Achieving high resolution and uniformity is crucial for the precise patterning of OLED devices’ anode, cathode, emissive layers, and other materials. Traditional methods like photolithography and inkjet printing have limitations in resolution and consistency. To overcome this, exploring alternative techniques such as nano-imprint lithography and laser-induced thermal imaging offers improved precision and uniformity. Cost and scalability are important considerations, especially for large-area displays. Traditional methods can be expensive and time-consuming, leading researchers to investigate more scalable and cost-effective options such as roll-to-roll gravure printing. The alignment of multiple layers in OLED devices poses challenges for patterning processes. Accurate alignment is crucial for optimal device performance, and novel techniques like polymer brushing can be developed to achieve precise layer alignment. Overcoming these challenges is essential to enhance OLED patterning techniques and advance the manufacturing of high-performance OLED displays. Advancements in fabrication processes, such as integrating atomic layer deposition (ALD) technology with plasma surface treatment, have been crucial. This progress, outlined in the research on red micro-LEDs, highlights promising developments, especially achieving larger mesa widths as small as 5 µm [240]. Utilizing plasma-enhanced chemical vapor deposition (PECVD) and ALD technologies for sidewall passivation and protection contribute to enhanced performance. The study also evaluates the lifetime of these micro-LEDs through accelerated burn-in processes and explores the application of pulse-width modulation (PWM) for driving micro-LEDs in display modules, aiming to reduce average power consumption.

### 6.3. Driving Circuits

OLED driving circuits pose challenges regarding current nonlinearity, temperature dependence, aging and degradation, and power consumption. The nonlinear current–voltage characteristic of OLEDs makes it difficult to achieve uniform current delivery, but active matrix organic light-emitting diodes (AMOLED) with thin-film transistors (TFTs) can show promise in addressing this issue. Temperature fluctuations affect OLED performance, necessitating temperature-compensated driving circuits that can play a vital role in overcoming this issue. Aging and degradation can impact OLEDs over time, but adaptive biasing systems can be useful to counteract these effects. Power consumption is another concern; there exist low-power solutions such as using digital-to-analog converters (DACs). These challenges drive the need for advancements in OLED driving circuit design to enhance their performance and efficiency. A world-first achievement integrates Si CMOS and scaled OSFET devices to create a high-pixel-density OLED display panel, demonstrating a monolithic structure suitable for versatile, low-power displays and next-generation XR devices [241].

### 6.4. Lifetime and Device Stability

OLED displays’ longevity and consistent performance are vital in the competitive flat-panel display (FPD) market. Ensuring optimal functioning and display quality over an extended period not only satisfies customers but also builds trust and credibility. The life of OLED displays depends on the stability of organic materials, the efficiency of the light-emitting process, and the impact of environmental conditions. Several key factors contribute to device stability and longevity. One significant concern is materials degradation, as the organic layers of OLEDs can deteriorate over time due to exposure to oxygen, moisture, and other environmental elements. This degradation can lead to diminished brightness and color shift, affecting device performance. Developing various encapsulation strategies to protect OLEDs from external influences is a hot technique nowadays. Giebink et al. introduced a framework with rate equations for charges, excitons, and defects within the PHOLED emissive layer (EML). This highlighted the critical impact of even a small percentage of defects on luminance reduction and suggested that a uniform exciton distribution could reduce triplet–triplet annihilation (TPA) and improve PHOLED lifetime [38]. Blue OLEDs utilizing triplet–triplet annihilation (TTA) up-conversion materials present a potential solution for extended lifetimes. However, a comprehensive understanding of exciton dynamics in degradation mechanisms is essential. Researchers established a numerical model of exciton dynamics to investigate the stability of blue OLEDs doped with TTA up-conversion hosts. Transient electroluminescence experiments reveal intrinsic parameters related to the TTA up-conversion process during aging, highlighting the main degradation mechanism as the damage to TTA materials by excess electrons in the emitting layer [145]. Experiments by various research groups confirmed the role of TPAs in PHOLED degradation [242]. The importance of achieving a broad and uniform exciton distribution to reduce TPA frequency and improve PHOLED lifetime was emphasized [243]. Inspired by this work, Lee et al. proposed a model involving a “manager” dopant to protect EML components against highly excited states, contributing to the comprehensive understanding of PHOLED degradation [244]. In studying the degradation mechanisms of phosphorescent OLEDs (Ir(ppy)3) and TADF OLEDs (4CzIPN) doped in an exciplex-forming cohost, researchers uncovered insights into the challenges faced by these devices. For Ir(ppy)3-based OLEDs, triplet–triplet-annihilation leads to the formation of defects causing a loss in luminance during device aging. On the other hand, 4CzIPN-based OLEDs face challenges from electron trapping-induced triplet–polaron annihilation and a narrow emission zone, significantly impairing device stability [245].

Thermal stability is another critical aspect, as high working temperatures can cause degradation of the organic layers, resulting in decreased device performance and lifetime. To enhance thermal stability, the use of thermally stable materials can improve device topologies. Maintaining a balanced distribution of charge carriers (electrons and holes) in OLEDs is crucial to avoid accumulating trapped charges, destabilizing the device, and shortening its lifetime. Mixed host materials can improve charge carrier balance and enhance charge injection layers. Device architecture also plays a role in OLED stability and longevity. For instance, adjusting the thickness of the emissive layer can impact device stability and efficiency. Different device topologies can be useful in achieving an optimal balance between stability and efficiency. By addressing materials degradation, thermal stability, charge carrier balance, and device architecture, researchers aim to enhance the stability and longevity of OLED displays in the competitive market.

In conclusion, implementing OLED technology faces significant challenges related to stability, lifetime, manufacturing, and driving circuits. However, researchers have made remarkable progress in addressing these issues through advancements in charge carrier balance, encapsulation techniques, materials selection, and device design. Efforts to commercialize OLEDs involve exploring novel materials, manufacturing methods, and eco-friendly approaches. Additionally, improvements in OLED driving circuits, including addressing nonlinearity, temperature dependence, aging, and power consumption, have been achieved by developing active matrix driving circuits, temperature-compensated systems, adaptive biasing techniques, and low-power digital-to-analog converters. Utilizing hybrid organic–inorganic perovskite materials and interdisciplinary research is promising for enhancing efficiency and stability in OLED driving circuits. Overcoming challenges in OLED patterning techniques, such as high-resolution and uniform patterning, material compatibility, and alignment, is crucial for advancing OLED technology. Researchers actively pursue innovative strategies like polymer brushing, inkjet printing, roll-to-roll gravure printing, and laser-induced thermal imaging.

The ongoing efforts in research and development aim to improve OLED performance, enhance commercial viability, and refine patterning techniques. As these challenges are addressed and resolved, OLED technology has the potential to revolutionize the lighting and display industries, providing long-lasting and high-quality displays that meet the demands of a competitive market.

## 7. Conclusions and Prospects

OLEDs have attracted much attention since they outperform more traditional display technologies like liquid crystal displays in terms of energy efficiency, color gamut, and contrast ratio. In this review paper, we have investigated the numerous causes of OLED deterioration and suggested remedies to prolong OLED lifetime. Also, we have emphasized the cutting-edge analytical methods applied to comprehend OLED degradation mechanisms and find solutions to extend their lifetime.

The slow deterioration of the organic elements used in manufacturing an OLED panel affects how long it will last. There are two basic categories of this degrading process: chemical degradation and electrical degradation. Organic materials undergo chemical degradation due to oxygen and moisture interacting with them, forming reactive species that can harm the OLED structure. On the other hand, the accumulation of trapped charges in the OLED layers results in electrical deterioration, which can reduce brightness and color accuracy. The use of subpar materials in OLED production is one of the leading causes of their degeneration. OLED materials are frequently expensive and challenging to produce, which might cause cost-cutting strategies that degrade the display’s overall quality. Moreover, using impure materials may result in structural flaws in OLEDs, which may speed up the deterioration process.

Environmental elements like oxygen and moisture are another factor that accelerates OLED degradation. Moisture and oxygen can react with the organic ingredients in OLEDs to generate reactive species, which can harm the structure of the device. OLEDs are extremely sensitive to these elements. Encapsulation techniques have been developed to solve this problem by creating a layer of protection between the OLED structure and the outside world. Although these encapsulation methods can significantly lengthen the life of OLED panels, they also raise production costs. OLED degradation is primarily influenced by electrical degradation as well. This kind of degradation, which can result in a loss of brightness and color accuracy, is brought on by the accumulation of trapped charges in the OLED layers. To solve this problem, several compensating methods have been reported that can lessen the negative effects of trapped charges on OLED performance. Using compensating voltage is one method that might diminish the impact of trapped charges on OLED performance.

Modern analytical methods have been influential in understanding the mechanics of OLED degradation and pinpointing strategies for extending their lifetime. One such method that has been utilized to research the chemical and electrical degradation pathways in OLEDs is time-resolved photoluminescence spectroscopy (TRPL). TRPL enables analysis of the degradation mechanisms by measuring the emission lifetime of excited states in OLED materials. For example, TRPL research has demonstrated that chemical deterioration is more likely to happen in the emissive layer than in the charge transport layers, which can aid in developing more effective encapsulation methods. X-ray photoelectron spectroscopy (XPS) is another crucial analytical method that can reveal details about OLED materials’ chemical makeup and electronic structure. The phenomena of chemical degradation in OLEDs are accessed by XPS analysis, which can assist in locating impurities and defects in OLED materials that may lead to degradation. For instance, the XPS study has demonstrated that reactive species, which can harm the OLED structure, can be created when contaminants like copper react with oxygen and moisture. In addition to these methods, scanning probe microscopy (SPM) has been utilized to investigate the shape and characteristics of OLED materials at the nanoscale. Scanning tunneling microscopy (STM) and atomic force microscopy (AFM) are examples of SPM techniques that can provide information.

## Data Availability

The data that support the findings of this study are available from the corresponding authors upon reasonable request.
